# Outcome of Early Cranioplasty in Trephine Syndrome or Paradoxical Brain Herniation: A Case Report and Literature Review

**DOI:** 10.7759/cureus.80922

**Published:** 2025-03-20

**Authors:** Zarbakhta Ashfaq, Hamza Ahmed, Adnan Khan, Aisha Mufti

**Affiliations:** 1 Surgery, Northwest General Hospital & Research Centre, Peshawar, PAK; 2 Surgical Intensive Care Unit, Northwest General Hospital & Research Centre, Peshawar, PAK; 3 Neurosurgery, Northwest General Hospital & Research Centre, Peshawar, PAK; 4 Critical Care, Northwest General Hospital & Research Centre, Peshawar, PAK

**Keywords:** cranioplasty, decompressive craniectomy, global aphasia, seizures, sinking skin flap syndrome

## Abstract

Sinking skin flap syndrome (SSFS) is a rare complication observed in patients after craniectomy. The intracranial pressure is decreased after craniectomy, resulting in some conditions. These include mental change, focal deficits, headache, seizures, and dysautonomia. Cranioplasty is the most commonly used treatment method for SSFS. Here, the case of a patient is presented with symptoms, including sudden loss of consciousness, aphasia, and right-side weakness. A computed tomography (CT) scan revealed hemorrhagic conversion of parietotemporal infarct with midline shift. A decompressive craniectomy was performed, and the patient developed SSFS later. Subsequently, the patient underwent cranioplasty and showed improvements in neurological deficits. SSFS is rare; only a few cases have been reported in the literature.

## Introduction

Sinking skin flap syndrome (SSFS), also known as Trephined syndrome, is a rare condition characterized by intracranial pressure falling below atmospheric pressure, leading to neurological dysfunction. It is most commonly observed as a complication of decompressive craniectomy, where a large portion of the bone flap is removed, altering intracranial dynamics. This procedure effectively converts the cranium from a closed system to an open cavity, disrupting normal cerebrospinal fluid circulation and brain homeostasis [1]. Patients with this syndrome may present with a range of neurological symptoms, including headache, dysautonomia, cognitive disturbances, seizures, and focal neurological deficits. In severe cases, the syndrome can progress to paradoxical brain herniation, where external atmospheric pressure exceeds intracranial pressure, potentially resulting in coma and death [2].

Early clinical recognition of SSFS is essential to prevent irreversible neurological damage. A high index of suspicion during hospitalization can facilitate timely intervention, mitigating the effects of the pressure shift from the atmosphere into the cranial cavity. Initial management focuses on restoring intracranial pressure, while definitive treatment involves cranioplasty - the surgical closure of the cranial defect using bone or mesh reconstruction.

Recovery following cranioplasty varies; while some patients experience rapid neurological improvement within 24 hours, others may take several weeks to months for full functional recovery [1]. Given the unpredictable course of recovery, close clinical monitoring is crucial in post-craniectomy patients.

Healthcare providers should maintain a high level of vigilance when evaluating post-craniectomy patients who develop new-onset neurological symptoms. Routine physical examinations play a critical role in assessing the development of a sunken skin flap. However, not all patients with visible cranial depression exhibit symptoms. A study reviewing 83 cases of SSFS found that all patients had a depressed scalp contour, but some were asymptomatic and diagnosed incidentally on imaging. Conversely, others exhibited motor, sensory, or cognitive impairments, which prompted further investigation and subsequent diagnosis [3].

This case report presents a patient who developed neurological deterioration within two weeks post-decompressive craniectomy, an earlier onset than typically reported. The patient's neurological deficits improved when transitioning from an upright to a supine position, further supporting the diagnosis of SSFS. While cases of sunken flaps following craniectomy have been documented, this case is unique due to its rapid onset and characteristic postural neurological changes.

## Case presentation

**Figure 1:**
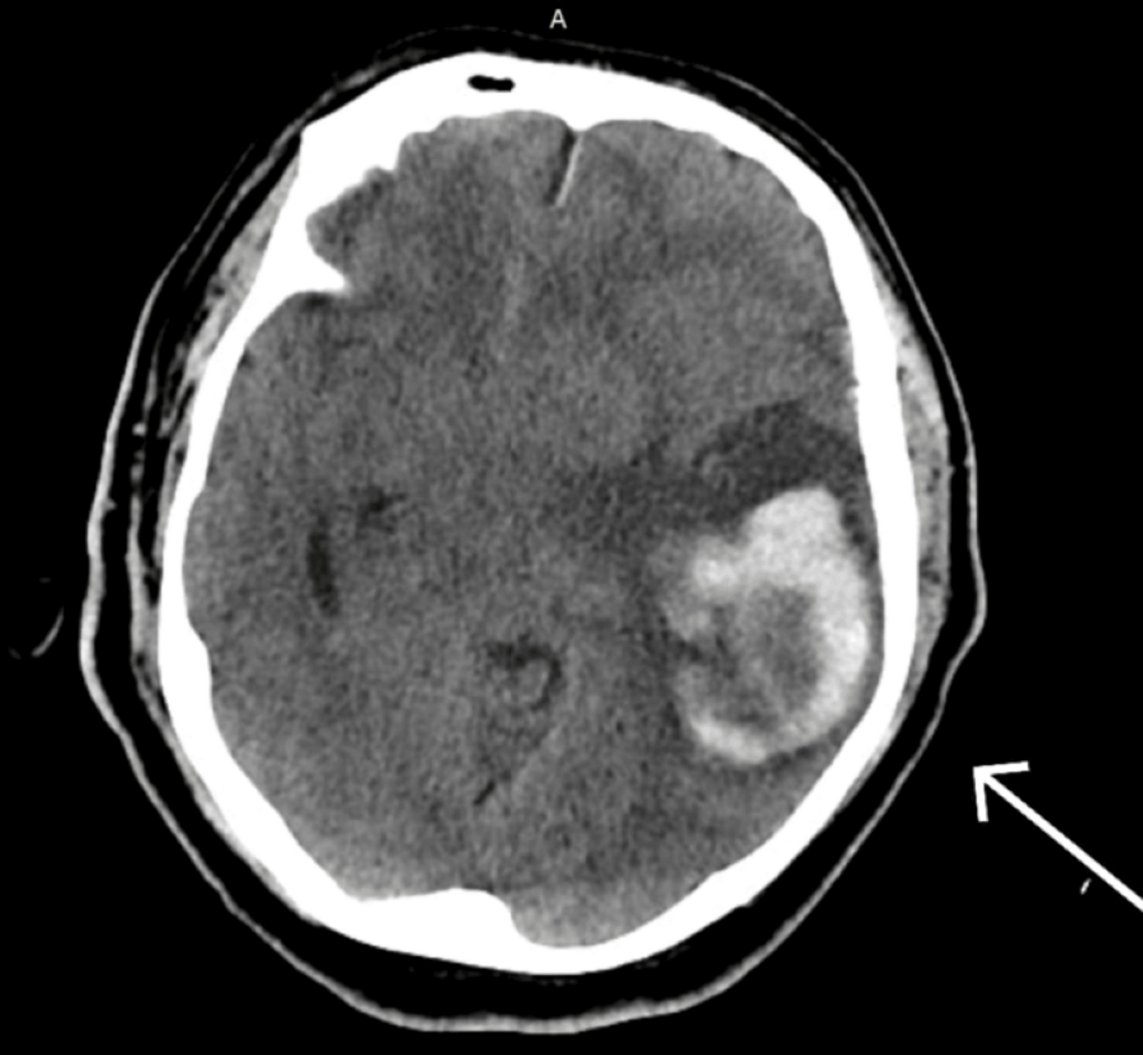
Initial presentation CT brain, with the arrow representing hemorrhagic area.

A 50-year-old known hypertensive male patient presented with chief complaints of sudden loss of consciousness, aphasia, and weakness on the right side. On arrival, his Glasgow Come Score (GCS) was 7/15. The patient was intubated; a computed tomography (CT) scan of the brain was done, which showed hemorrhagic conversion of parietotemporal infarct with midline shift (Figure 1).

**Figure 2:**
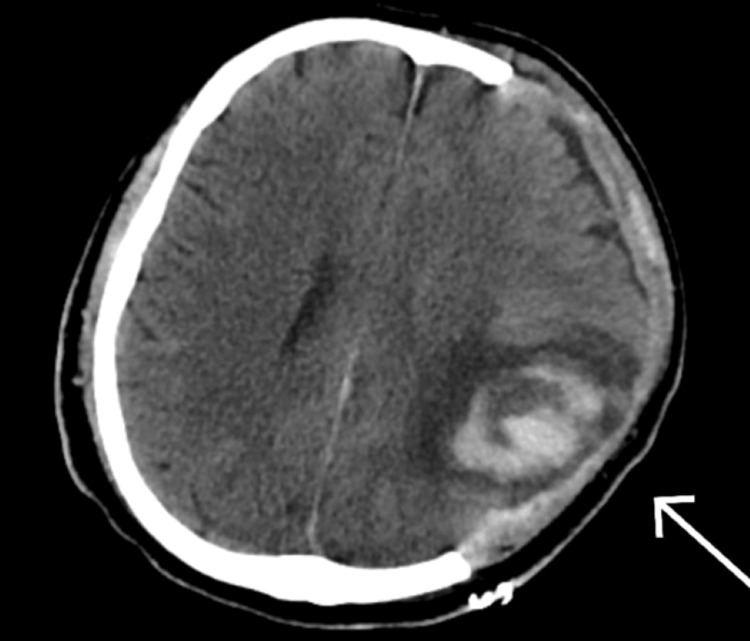
CT brain post-operatively, with the arrow representing post-craniectomy changes, as mentioned in the text.

He was admitted for decompressive craniectomy. Post-surgery, the patient was monitored in the surgical intensive care unit and was transferred after extubation and tracheostomy to the floor with a GCS of 10/10t. In the ward, GCS dropped to 3/10t. The patient was then shifted back to the intensive care unit, and magnetic resonance imaging (MRI) and CT brain showed post-craniectomy changes on the left side of the calvarium with underlying subdural fluid collection (Figure 2). 

**Figure 3:**
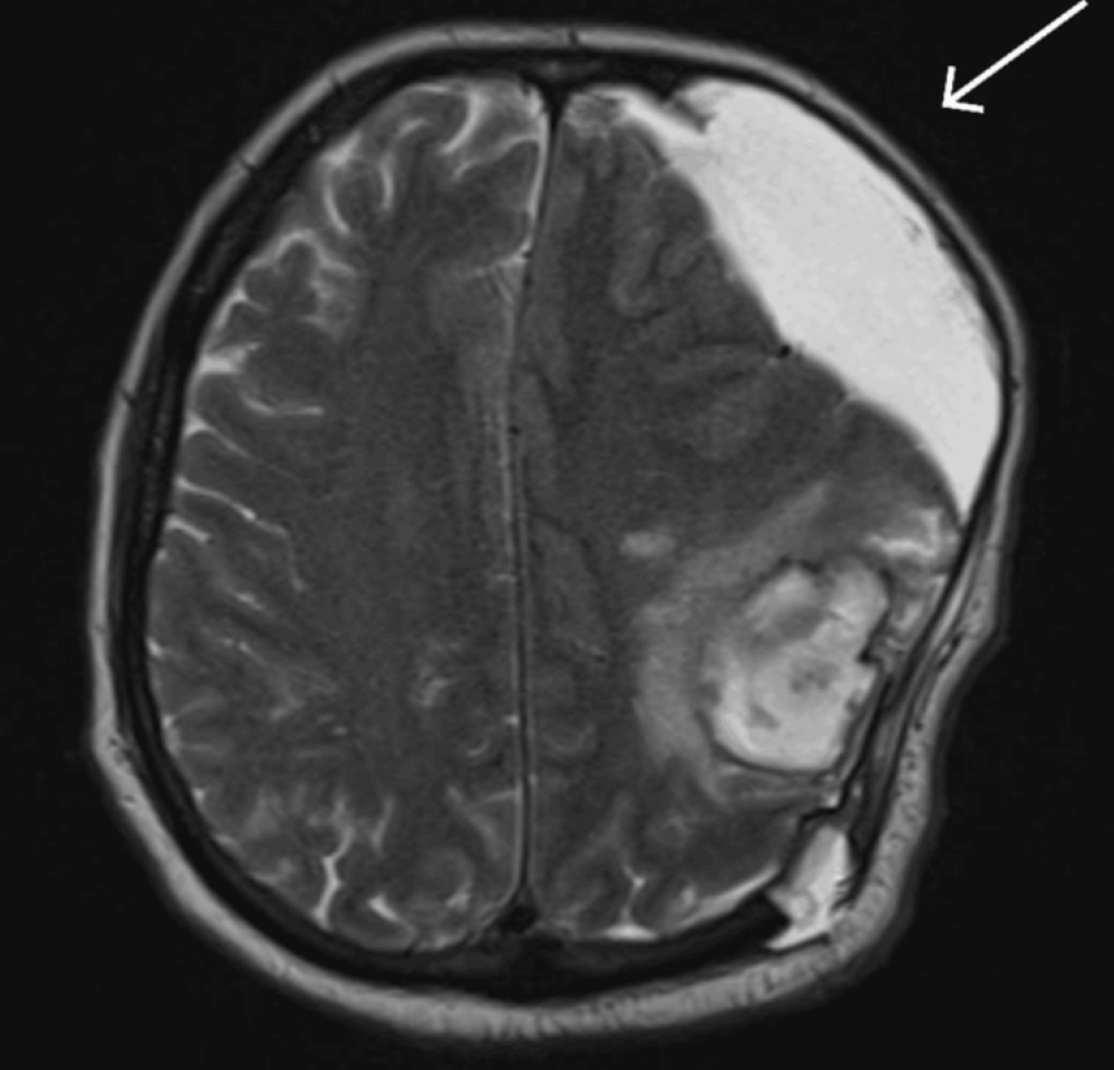
MRI T2-weighted images showing post-op changes, with the arrow pointing towards the hematoma.

MRI done showing heterogeneous signal intensity area in the left frontoparietal lobes has signal characteristics suggestive of hematoma (with blood of varying ages) (Figure 3). Significant edema resulted in a midline shift, parafalcine, and uncal herniation.

**Figure 4:**
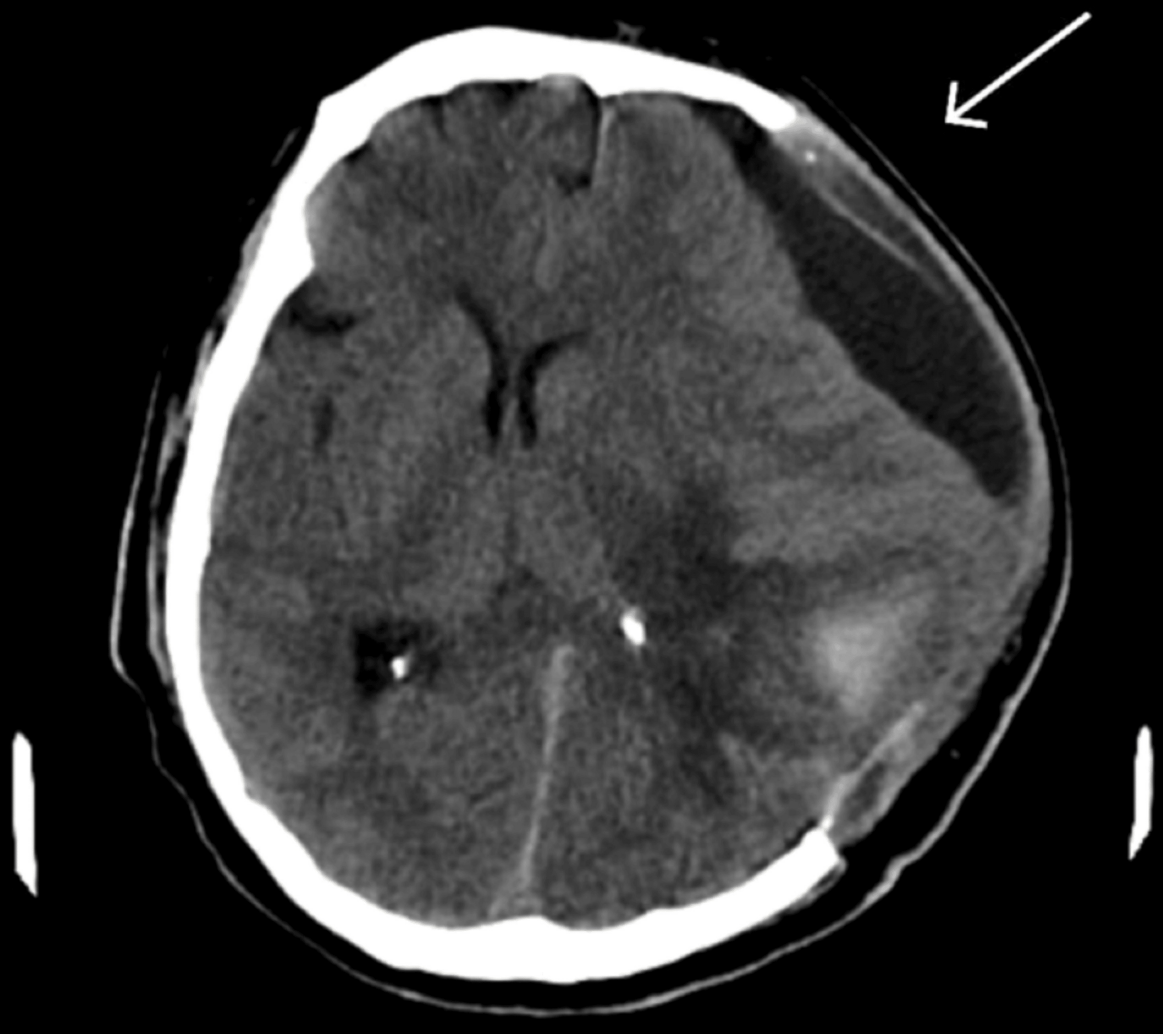
CT brain, with the arrow pointing towards hygroma.

The CT scan conducted in a supine position posed limitations in accurately assessing the patient’s condition, particularly regarding the detection of sinking skin syndrome (Figure 4). Due to the patient's lying down position, the imaging did not effectively capture the characteristic inward depression of the skull that accompanies sinking skin syndrome. Instead, the scan predominantly revealed the presence of a hygroma, accompanied by a midline shift. This midline shift indicated a significant mass effect, likely related to the hygroma, but it obscured any potential changes in the skull shape that might have suggested sinking skin syndrome. Consequently, the reliance on the CT findings alone may have led to an incomplete clinical picture, emphasizing the need for a comprehensive assessment that includes both imaging and clinical evaluation to fully understand the patient’s condition and guide appropriate management.

In this case, the patient was admitted to the surgical intensive care unit (ICU) with fluctuating GCS scores ranging from 10 to 12, a concerning sign of altered consciousness. Notably, the patient’s GCS demonstrated a marked decline when in an upright position, which was promptly reversed upon lying flat. This phenomenon occurred within two weeks following a decompressive craniectomy, raising suspicions about possible complications related to the surgery.

Initially, subclinical seizures were considered a differential diagnosis due to the fluctuations in consciousness; however, electroencephalogram (EEG) monitoring failed to demonstrate any seizure activity. In parallel, a thorough infectious workup was conducted, which identified Enterobacter species in sputum cultures. Despite this finding, the clinical picture did not support sepsis, as the patient's vital signs remained stable throughout the evaluation.

A complete sepsis workup was undertaken, including complete blood count and lactate level measurements, along with repeat sputum cultures to rule out any infectious processes. Concurrently, urine electrolytes were assessed to check for any imbalances that could affect neurological function. Additionally, tests for diabetes insipidus were performed, involving serum and urine osmolality measurements to evaluate the patient’s fluid balance and endocrine function.

Considering the potential for pulmonary complications, the team also evaluated the patient for secretions or aspiration pneumonia, which could contribute to respiratory distress and subsequently affect consciousness. Despite these extensive investigations, the underlying reason for the fluctuating GCS remained elusive until the diagnosis of sinking flap syndrome was suggested. This condition, characterized by a reduction in intracranial pressure due to the absence of the skull in the area of the craniectomy, can lead to neurological deficits and fluctuating mental status.

In light of this diagnosis, the neurosurgery and surgical ICU teams reached a consensus recommending surgical intervention to address the sinking flap syndrome. The following are the results of investigation reports, for which he was ruled out for other causes. Laboratory tests assessed the patient’s metabolic status and potential infection. The hemoglobin level was below normal (10.40 g/dL). The white blood cell differential indicated an increase in the neutrophils (90%). An increase in the neutrophils reflects an acute inflammatory response or infection. On the other hand, the monocyte and lymphocyte counts were low (4% and 5%, respectively), implying an underlying immunocompromised state. The hematocrit (HCT) was found below average (30.80%). It was an indication of possible postoperative anemia or dilutional effects.

The procalcitonin (PCT) levels are high (1.35 ng/mL). PCT is a biomarker that is often associated with bacterial infection. Here, PCT supported the suspicion of sepsis. The renal function was noted to exhibit a high glomerular filtration rate. The high eGFR (2.16.82 mL/Min/1.732) suggested hyperfiltration due to the volume status changes. C-reactive protein (CRP) levels were within normal limits, which indicated a chronic rather than an ongoing or acute inflammatory state. Urine osmolality was within normal limits, while serum osmolality was high. The high serum osmolality (316 mOsmol/kg) indicated a potential hyperosmolarity. The discrepancy raises concerns about renal concentrating ability or excessive serum solute loads.

**Figure 5:**
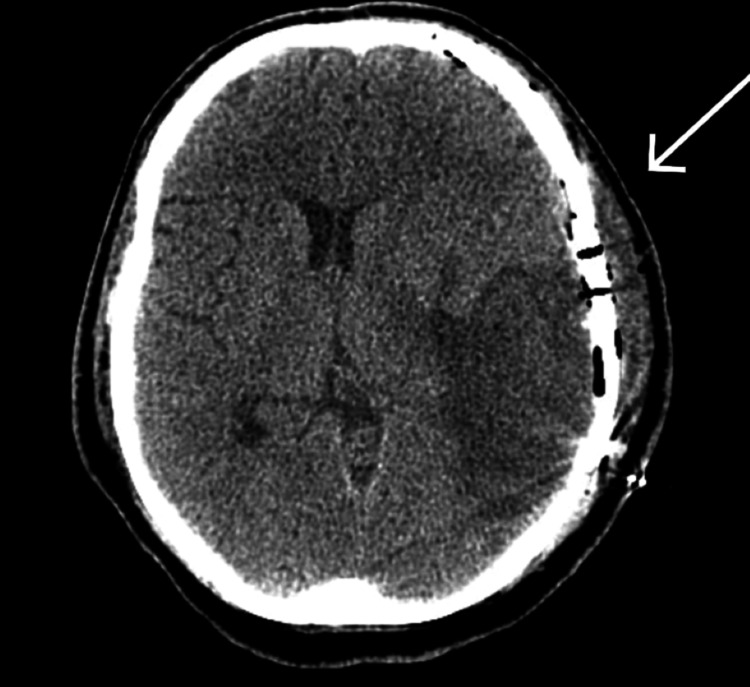
Post-cranioplasty CT brain, with the arrow representing distinct improvements of the lesion, and a decreased midline shift in the image is also appreciable.

The serum culture yielded growth of Klebsiella oxytoca and Enterobacter spp. after 24 hours of aerobic incubation at 37°C and exhibited resistance to all the tested antibiotics. The patient was taken in cranioplasty, and he was monitored after surgery. His GCS on the first day post-operation improved to 14/15. His repeat CT showed a decrease in midline shift and improvement. He was monitored in the ICU for two days and shifted to the ward. His neurological dysfunction in an upright position was not present anymore, and his head end was kept up. A drastic improvement was seen in his previous neurological deficit. His post-cranioplasty CT is shown in Figure 5.

## Discussion

Decompressive craniectomy is a surgical procedure in which a bone flap is removed to reduce intracranial pressure (ICP) in patients with conditions such as stroke or traumatic brain injury (TBI) [4]. The most common indications for decompressive craniectomy include malignant middle cerebral artery infarction, subarachnoid hemorrhage, and severe TBI. In this case, the procedure was performed due to hemorrhagic conversion of a parietotemporal infarct with a midline shift observed on the CT scan. Several studies indicate that decompressive craniectomy is a life-saving intervention, improving survival rates and neurological outcomes, particularly when performed in the early stages of the disease [5-8].

A large fronto-temporoparietal craniectomy (≥15 cm in diameter) is often preferred, as it enhances surgical efficacy and minimizes external cerebral herniation. Early cranioplasty, performed as soon as brain relaxation is achieved, may help reduce long-term complications associated with decompressive craniectomy [9]. The complications of craniectomy are typically categorized into early (within four weeks) and late (beyond four weeks). Early complications, which often occur during hospitalization, can be anticipated and managed promptly. Kurland et al. [10] classified these complications into hemorrhagic, infectious/inflammatory, and cerebrospinal fluid (CSF) disturbances.

Several studies highlight the early complications of decompressive craniectomy, including cerebral contusion expansion, new-onset subdural or epidural hematoma contralateral to the craniectomy site, epilepsy, CSF leakage, and external cerebral herniation, most of which occur within the first week post-operatively. In contrast, subdural effusions and post-operative infections tend to emerge between one and four weeks, while syndrome of the trephined (SSFS) and post-traumatic hydrocephalus usually develop after one month.

SSFS, also known as the "syndrome of the trephined," is a rare but significant delayed complication of craniectomy. It was first described by Grant et al. in 1939, who reported symptoms such as discomfort at the surgical site, cognitive dysfunction, dizziness, headache, and fatigue [11]. Yamamura et al. later expanded on the syndrome in 1977 [12]. Yang et al. observed SSFS in 13% of patients undergoing decompressive craniectomy, with symptom onset ranging from 28 to 188 days post-surgery [13].

A systematic review by Ashayeri et al. [14] found that SSFS can affect individuals of all age groups, with a higher prevalence in males (60%). The onset of symptoms is highly variable, though motor deficits and cognitive decline are the most commonly reported. While some patients develop headaches, these are less frequent. Interestingly, most patients experience symptom improvement within 24 hours of cranioplasty, supporting its role as the definitive treatment [14].

The pathophysiology of SSFS remains incompletely understood. One hypothesis suggests that removing a large skull segment converts the cranial vault from a "closed box" to an "open system," disrupting normal intracranial dynamics. The lack of bone protection, combined with atmospheric pressure and gravitational forces, leads to paradoxical brain herniation and neurological deterioration. Langfitt [15] proposed that atmospheric pressure directly transmits forces to the intracranial cavity, causing an inward collapse of the scalp over the craniectomy site.

Clinically, patients with SSFS often present with new-onset neurological deficits, including headache, dizziness, cognitive decline, mood disturbances, and focal neurological signs. Some individuals also develop dysautonomic symptoms, such as postural hypotension, bowel or bladder dysfunction, and fatigue. Characteristic imaging findings include a depressed skin flap, midline shift, paradoxical herniation, and distortion of intracranial structures [16]. In the present case, our patient exhibited hematoma formation, brain edema, parafalcine and uncal herniation, and a significant mass effect on the brainstem.

Several published case reports parallel our findings. Khan et al. [2] described a patient who developed SSFS one year after craniectomy for TBI. Initially presenting with GCS 10/15, the patient’s condition deteriorated (GCS 8/15), with a subsequent CT scan confirming SSFS. Although cranioplasty was recommended, the family deferred, leading to temporary ICP elevation via VP shunt adjustment, which improved symptoms within five days. Jeyaraj [17] reported another case of SSFS occurring three months post-craniectomy, where rapid neurological decline necessitated urgent cranioplasty, leading to marked cognitive and motor recovery. Similarly, Park et al. [18] documented two cases of SSFS, both of which showed significant improvement following cranioplasty.

Currently, cranioplasty remains the gold-standard treatment for SSFS, as it restores normal CSF dynamics, ICP, and cerebral perfusion. Our patient demonstrated neurological recovery post-cranioplasty, with a repeat CT scan confirming reduced midline shift and reversal of paradoxical herniation. Similar improvements have been reported in the literature, with Sakamoto et al. [19] documenting pre- and post-cranioplasty CT scans demonstrating clear radiological improvements.

The optimal timing of cranioplasty remains debated. Traditionally, surgeons delay cranioplasty three to six months post-craniectomy to allow for brain relaxation and wound healing. Some studies suggest late cranioplasty is superior, with Xu et al. [20] reporting no significant functional advantage of early cranioplasty, despite its shorter hospital stay. Salma et al. [21] also favored delayed cranioplasty, citing reduced surgical complications.

Conversely, evidence supporting early cranioplasty continues to grow. Kim et al. [22] found that early cranioplasty significantly improved cognitive and functional recovery, particularly in mobility, language, and self-care. Tasiou et al. [23], in a systematic review, concluded that early cranioplasty may offer better neurological recovery in select cases. Similarly, Annan et al. [3] emphasized early intervention in SSFS to prevent further deterioration. Ultimately, the decision between early and late cranioplasty should be individualized. In cases with midline shift or paradoxical herniation, early cranioplasty is preferred, as suggested in Park et al.'s study [18]. A notable aspect of this case is the presence of a large hygroma, which initially complicated the diagnosis. SSFS can co-exist with hygromas, leading to distinct cranial morphological changes. While hygromas typically cause outward skull bulging due to CSF accumulation, SSFS is characterized by an inward depression of the scalp.

Our multidisciplinary team initially considered needle aspiration of the hygroma to relieve ICP. However, the literature review indicated a high risk of exacerbating SSFS. Needle tapping may provide temporary symptomatic relief, but it can compromise structural integrity, leading to worsened neurological deficits and aesthetic concerns. Instead, we recommended bone repositioning, which not only addressed the hygroma but also mitigated the risk of SSFS, ultimately leading to a favorable surgical outcome.

## Conclusions

SSFS is a serious and often overlooked delayed complication of craniectomy, requiring high clinical suspicion in post-surgical patients presenting with new-onset neurological deterioration and dysautonomic symptoms. Although SSFS is typically considered a late-onset complication, our case highlights an early presentation within two weeks of craniectomy, emphasizing the need for prompt recognition and intervention. This case report underscores the complexity and diagnostic challenges of SSFS, reinforcing the importance of comprehensive clinical evaluation, including laboratory assessments and imaging, to rule out alternative causes and confirm the diagnosis.
